# Forensic age estimation: comparison and validation of the Iscan method in 3D reconstructions using a surface scanner in a Spanish population

**DOI:** 10.1007/s00414-023-02983-6

**Published:** 2023-03-17

**Authors:** Cristina M. Beltran-Aroca, Manuel Lopez-Alcaraz, Pablo Perez-Jorge, Jose L. Velazquez-Gomez, Pilar Font-Ugalde, Eloy Girela-Lopez

**Affiliations:** 1 Section of Legal and Forensic Medicine, Faculty of Medicine and Nursing, University of Cordoba, Av. Menendez Pidal S/N. 14004, Cordoba, Spain; 2 Present Address: Institute of Legal Medicine and Forensic Sciences of Cordoba, Cordoba, Spain; 3 Department of Medical and Surgical Science, Faculty of Medicine and Nursing, University of Cordoba, Maimonides Biomedical Research Institute of Cordoba (IMIBIC), Cordoba, Spain

**Keywords:** Age estimation, Surface scanner, Forensic anthropology, Virtual anthropology, Rib, Forensic medicine

## Abstract

When investigating a death, post-mortem identification provides with results of great legal and humanitarian significance. The effectiveness of the methods used to estimate age depends on the reference population, considering variables such as sex and ancestry. The aim of this study was to validate the Iscan method to estimate age in a Spanish forensic population, comparing the estimates obtained in dry bones and 3D reconstructions created with a surface scanner. We carried out a cross-sectional study on 109 autopsied corpses (67% male), scanning the sternal end of the right fourth rib in a 3D mesh, using an EinScan-Pro® surface scanner (precision: 0.05 mm). Two observers estimated the phases in dry bones and 3D images according to the Iscan method and to the sex of the subject. The mean age was 57.73 years (SD = 19.12 years;18–93 years). The intra-observer agreement was almost perfect in bones (*κ* = 0.877–0.960) and 3D images (*κ* = 0.954), while the inter-observer agreement was almost perfect in bones (*κ* = 0.813) and substantial in 3D images (*κ* = 0.727). The correlation with the Iscan phases was very strong in bones (Rho = 0.794–0.820; *p* < 0.001) and strong in 3D images (Rho = 0.690–0.691; *p* < 0.001). Both sex-adjusted linear regression models were significant (dry bones: *R*^2^ = 0.65; SEE =  ± 11.264 years; 3D images: *R*^2^ = 0.50; SEE =  ± 13.537 years) from phase 4 onwards. An overestimation of age was observed in the first phases, and an underestimation in the later ones. Virtual analysis using a surface scanner in the fourth rib is a valid means of estimating age. However, the error values and confidence intervals were considerable, so the joint use of different methods and anatomical sites is recommended.

## Introduction

### Forensic identification

During the medical-legal investigation of a death, the post-mortem identification process is carried out in an integrated way. Its results have great legal and humanitarian importance both when major catastrophes occur and in routine forensic practice [1]. One of the top priorities is the identification of human remains, which must be carried out using a strict scientific-technical procedure to allow their identity to be established accurately. Positive identification is only possible in a case when the ante-mortem and post-mortem data match sufficiently closely to conclude that they are from the same individual, excluding all other reasonable possibilities [2]. When carrying out subject identification, the INTERPOL protocols draw a distinction between primary techniques (friction ridge, dentistry or DNA analysis) and secondary techniques (medical information, tattoos, personal description, anthropology, effects found on the bodies of victims such as jewellery, articles of clothing, and personal identification documents). The former have a proven scientific value that will permit a positive identification to be made, while the latter can further strengthen a general circumstantial identification [3]. When identification cannot initially be made and there is no circumstantial evidence, it is recommended to evaluate the biological profile of the subject using anthropological analysis [4]. The individual’s biological information individual which is analysed includes age, sex, ancestry, and stature, in order to obtain a provisional identification which meets the legal requirements [5]. Of all the parameters contained in the profile, the estimation of a subject’s age is of maximum importance. In the case of unidentified human remains, age at death allows us to narrow down the list of potential missing persons and can relate the estimated age of a possible candidate to their age at the time of disappearance [6].

### Estimation of age

The current social and political situation has led the scientific community to coordinate and standardize the methods and procedures used for estimating age, and update them periodically through protocols, reviews, and recommendations [7]. A report by the Scientific Working Group for Forensic Anthropology (SWGANTH) on good practices in forensic case analysis states that estimation of age at death should be made “in a spirit of scientific integrity.” Subjective interpretation must be kept to a minimum, the techniques applied must be scientifically proven and appropriate to the case, and the limitations and weaknesses of each method must be explained [8]. The first step in age estimation is to assess physiological or biological age (effect of aging on the morphological aspects of bones) and then to attempt to correlate this with chronological age (length of a person’s life). Nevertheless, we must take into account the fact that throughout an individual’s life, a number of modifications occur to the skeleton due to the interaction of genetic, cultural, environmental factors, diseases, occupational stress, physical activities, or even the subject’s weight [9]. These changes generate biological variability, which makes it difficult to make a perfect match between the biological age and the chronological age, leading to a certain margin of error in age estimation, especially at advanced ages [10]. In fact, the effectiveness of the methods used to estimate age depends directly on the reference population, so it is necessary to consider variables such as sex and ancestry [11]. The measurements are carried out analytically and comparatively, reconstructing the biological characteristics of the individuals and their group in the fullest possible way, and an indicator for determining age is considered acceptable when it meets a series of criteria: (1) morphological changes occur unidirectionally with age, (2) the characteristics have a good correlation with the chronological age, (3) the changes occur at approximately the same age in all individuals, and (4) the features are measured with known intra- and inter-observer error rates [8].

In adults, categorical morphological methods have traditionally been used to analyse the degenerative changes in certain articular surfaces as the subject’s age increases, in areas such as the pubic symphysis [12], the auricular surface of the ilium [13], the 1st rib [14, 15], or the medial end of the 4th rib (costochondral junction) [16–18]. Of these, due to its location and function, the costochondral junction constitutes a highly stable area that allows any changes that may have occurred during the life of the individual to be observed without interference from biomechanical effects, pregnancy or childbirth [18]. Iscan et al. [16] analysed the morphological changes of the medial end of the 4th rib, which, due to an excess of endosteal resorption with respect to periosteal deposition, lead to a gradual deepening of the sternal joint and the thinning of its walls with increasing age [19]. Later, their initial components method [16] was superseded by a phase-based method, which is currently considered the reference method [17, 18]. In their study, Iscan et al. analysed first the right 4th rib from 118 Caucasian males between 17 and 85 years of age [17], and then from 86 females between 14 and 90 years old [18]. They published a descriptive scheme based on 9 different phases from 0 to 8, for each sex respectively, which were fundamentally based on characteristics such as depth and shape of the rib articulation, configuration of the rib edge and thickness of its walls, and the general condition of the rib. Previous work has suggested that the sternal end of the 4th rib exhibits less variation than the symphysis pubis and is associated with less inter-observer error [20, 21]. In addition, there is a lack of accuracy in pubic symphysis estimates in subjects over 50 years of age [22], which means that the rib is a more precise, consistent indicator in that age range [10, 23]. However, the fourth rib method has been widely questioned in relation to population variability [24, 25], bias, or reproducibility [26]. Due to these limitations, various adaptations have been put forward [27, 28] and multiple validation studies have been conducted in different populations [23, 29–34], although to date, no studies have been carried out in a Spanish population.

Parallel to the traditional method, other alternative complementary techniques have also been developed in ribs [35]. At the microstructural level, Jowsey applied quantification techniques to bone microradiographs and observed the predominance of bone resorption in older adults compared to subadult individuals [36]. Previous histological studies have also correlated age with certain bone microstructures [37], reporting good results in ribs [38]. However, this type of analysis requires the use of cross sections, which involves having to damage the bone.

Over recent years, the use of virtual anthropology techniques [39], in particular the technique known as virtual morphology [40], has spread. Clinical images from computed tomography (CT) or magnetic resonance imaging (MRI) have provided an excellent source of complementary data for non-invasive studies in modern populations [11], which have been successfully used to estimate age [41], specifically applying the Iscan method [42–45]. In addition, other technologies such as surface scanners have also been widely used to obtain high-resolution 3D models. These optical systems measure anatomical structures using visible light to generate dense point clouds and 3D polygonal meshes, with the added advantages of low cost, easier portability, optimal resolution, and fast post-processing [46]. They also allow researchers to create 3D replicas and share the images, via a server or on the Internet. Imaging is particularly useful for an easier and faster data exchange between institutions or the possibility of permanent documentation of skeletal remains and trauma, for illustrative, archival, and forensic purposes. Sharing images has enabled virtual skeletal collections to be compiled which would avoid physically travelling to take measurements and reference points for research [47]. The constant use of skeletal remains for research purposes interferes with their state of preservation, so image storage provides permanent access even if the actual remains are not available or no longer exist. In addition, this system has been used for several years now to determine sex or age in a biological profile, comparing different models and technologies to maximize their results. Thus, 3D models provide additional opportunities for data analysis using new variables, such as surface areas, volumes, contours, surface relief, and semi-landmarks [48, 49]. However, its application in the creation of 3D images of ribs has not been described to date.

For the first time, in this work, we have applied the method developed by Iscan to estimate age using the fourth right rib in a Spanish forensic population using a sample of dry bones and their 3D reconstructions obtained by surface scanner. The objectives were (1) to compare the estimates obtained in the two samples and (2) to validate the Iscan method through our results.

## Material and methods

### Design

In this cross-sectional, descriptive, observational epidemiological study, the objective was to validate the age estimation method using the 4th right rib both in dry bones and 3D images, and to determine the degree of agreement between the two modalities, in a forensic sample of the Spanish adult population.

### Study population

A total of 109 right fourth ribs were collected from autopsied cadavers at the Institute of Legal Medicine and Forensic Sciences of Córdoba (ILMFC-CO) (Spain). Samples were taken from corpses whose sex and age (minimum 18 years) were known, and samples were excluded where chest trauma to the anterior wall had occurred with involvement of the costochondral joint of the 4th right rib, in which the subject had a known history of chronic respiratory pathologies, pulmonary emphysema (or macroscopic presence of suggestive signs), asthma, or interstitial diseases or in cases of congenital malformations and/or lesions affecting the anterior chest wall.

### Procedure

During the autopsy, samples were extracted after locating the 4th right rib. For this, a 2.5-cm cut was made from the costochondral junction towards both the medial and the lateral sides. The maximum possible amount of soft tissue was removed from each rib, which was subsequently subjected to a maceration process for 10 days. The samples were then submerged in a mixture of water and sodium hexametaphosphate (2 mg per litre of water) at 90 °C until all the soft tissue and cartilage remains were completely removed. Finally, the samples were stored at room temperature until completely dry [50].

After the samples were treated, the sternal end of the rib was scanned in 3D meshes using an EinScan Pro structured light surface scanner (Shining 3D®, Hangzhou Xianlin 3D Technology Co., Ltd., China) consisting of two cameras. First, the scanner was calibrated manually, followed by a white balance calibration. Next, with the scanner in a fixed position on a tripod, the scan was performed in automatic mode on a turntable (20 turntable steps), with a precision of 0.05 mm per scan (dot distance 0.16 mm) for objects in a range of sizes between 30 and 150 mm. Once the image was obtained, the surplus elements were deleted, and the sample was meshed using a watertight model. The images were exported to STL format. The data obtained was processed using the MeshLab® program for the visualization and examination of scanned images (360° manipulation).

The sample was studied by two observers (both of them were M.D. and Ph.D.; Observer A, a specialist in legal and forensic medicine with a Master’s degree in Physical and Forensic Anthropology; Observer B, a forensic pathologist with a Master’s degree in Physical and Forensic Anthropology). Each observer made two independent blind observations on each type of sample (2 dry bone and 2 3D images), taken at least 15 days apart to avoid memory bias. The observations were performed following the phase descriptions proposed in the Iscan method based on sex [17, 18] and by comparison with France Casting® reference casts [51]. To carry out the classification of the sample in the different phases, the following characteristics were considered [17, 18]: pit depth and shape, configuration of the walls and rim, osteophytes, and the overall texture and quality of the bone (the latter only possible in the analysis of dry bones).

### Study variables

The following variables were analysed: Iscan phase estimated by the observers according to sex (1–8), Iscan phase related to chronological age according to sex (Iscan phase) (1–8), the subject’s sex, and chronological age.

### Statistical method

Firstly, a descriptive study was carried out in the qualitative variables by calculating counts (*n*) and proportions (%), and in the quantitative variables by calculating the mean (*m*), standard deviation (SD), median (me) and interquartile range (IQR), and minimum and maximum values (min–max). Confidence intervals were calculated using 95% certainty (95% CI).

In the bivariate analysis for continuous variables, we used the Kolmogorov–Smirnov to test their adjustment to a normal distribution, and the Mann–Whitney *U* test to analyse the age distribution according to sex. To estimate the degree of intra-observer, inter-observer, and inter-method agreement (dry bones/3D images), the maximum and minimum values of Cohen’s weighted Kappa index (*κ*) [52] were calculated, using the square weights matrix with the Landis and Koch scale [53]. The theoretical chi-square distribution was applied to compare the *κ* values, and a mean value was obtained. Next, the percentage agreement of the phases identified by the observers was calculated, both for 0-stage and 1-stage tolerance [54]. We used Wilcoxon’s signed-rank test to analyse the differences evaluated, and Spearman’s linear correlation coefficient Rho (*ρ*) to correlate the phases estimated by the observers and those of the Iscan method.

Finally, a sex-adjusted multiple linear regression analysis was carried out to predict the relationship between the predictor variables (estimated phases and sex) and the explained variables (actual age of the subject), using the adjusted *R*^2^ values, the standard error of the estimate (SEE), and standard error (SE) for each phase, in dry bones and 3D images. The 95% CI for individual age prediction according to the 4th rib phases were calculated using the bias-corrected and accelerated bootstrap method (BCa) (number of replicates *B* = 10,000).

All hypothesis contrasts were bilateral, and those with a *p* value < 0.05 were considered statistically significant. The data were collected in an Excel file and subsequently filtered, processed, and analysed using the PASW Statistics 28 (IBM-SPSS) and Epidat 4.2 statistical programs.

### Ethical considerations

This research study complied with the standards of good clinical practice and the principles established in the Declaration of Helsinki on biomedical research. Sample collection was carried out with the prior approval of the ILMFC-CO Teaching Commission and the Córdoba Research Ethics Committee (ref. no. 4184; 03/06/2019).

## Results

### Description of the sample

**Fig. 1:**
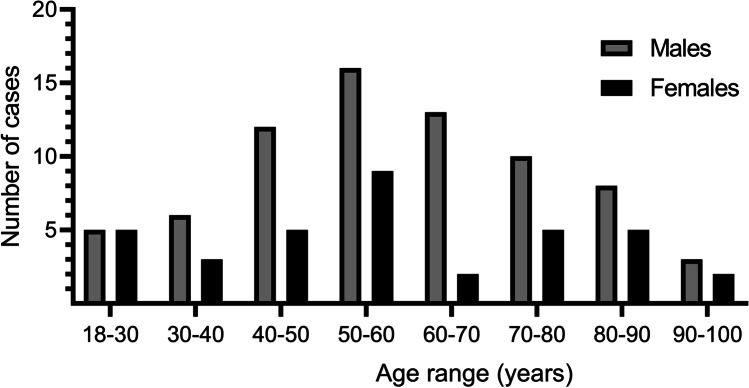
Distribution of the sample according to age and sex

Of the 109 cases analysed, 73 were male (67%), and 36 were female (33%). The mean age was 57.73 years (SD = 19.12; 95% CI 54.10–61.36), with a minimum of 18 and a maximum of 93 years. Figure 1 shows the distribution of the subject’s age according to sex, grouped by age intervals. The mean age in the male group was 58.41 years (SD = 17.86; 95% CI 54.24–62.58), with a range between 21 and 93 years, while in females, it was 56.36 years (SD = 21.66; 95% CI 49.03–63.69), with a range between 18 and 93 years. The age distribution did not differ significantly according to sex (*p* = 0.711).

### Intra-observer agreement for each method

In dry bones, there was almost perfect agreement between the first and second observation for Observer A, with Kappa (*κ*) = 0.877 (*p* < 0.001; 95% CI 0.809–0.944), and a value of *κ* = 0.960 for Observer B (*p* < 0.001, 95% CI 0.941–0.980).

When using the 3D images, Observer A also obtained an almost perfect agreement with *κ* = 0.954 (*p* < 0.001; 95% CI 0.920–0.987), while the value was *κ* = 0.954 (*p* < 0.001; 95% CI 0.929–0.978) for Observer B.

The repeatability of the estimates in each observer did not show statistically significant differences between their first and second observations, both in dry bones and in 3D images. Observer A’s estimates obtained a median of 6 (IQR = 2; *Z* =  − 0.2092; *p* = 0.771) in bones, and 7 (IQR = 2; *Z* =  − 0.037; *p* = 0.971) in 3D reconstructions, while for Observer B, the median was 6 for the two observations of dry bones (IQR = 3; *Z* =  − 1.225; *p* = 0.221) and the 3D images (IQR = 2; *Z* =  − 0.784; *p* = 0.433).

### Inter-observer agreement depending on the method

The agreement between observers for dry bones was almost perfect, while in the 3D images, it was substantial. Table 1 also shows the percentages with 0-stage and 1-stage tolerance. In both types of samples, the agreement trend was greater from phase 5 onwards, especially between phases 6 and 8. Significant differences were observed when comparing the distribution of the phases estimated by the two observers.

**Table 1:** Inter-observer agreement in dry bones and 3D images

	Cohen’s kappa						Percent agreement (%)		Wilcoxon test	
	Lowest *κ*	Highest *κ*	*p*	Mean ***κ***	95% CI	*p*	0-stage tolerance	1-stage tolerance	*Z*	*p*
Dry bones	0.810	0.817	< 0.001	0.813	0.756–0.871	0.905	56.9	90.8	− 3.287^a^	0.001
3D images	0.714	0.740	< 0.001	0.727	0.650–0.804	0.741	47.7	81.7	− 4.902^a^	< 0.001

^a^Based on positive ranges

### Inter-method agreement

The agreement between the observations performed with dry bones and 3D images for both observers was almost perfect. Table 2 shows the agreement of the observers when using both methods. No statistically significant differences were observed in the phase distribution performed by the observers in the two methods (Table 3).

**Table 2:** Observers agreement for dry bones and 3D images

Observer A	3D images									
Dry bones	Phases	1	2	3	4	5	6	7	8	Total
	1	0								0
	2		4							4
	3			0	1	1				2
	4				1	2				3
	5					10	4			14
	6					4	18	4	7	33
	7					1	3	5	1	10
	8					1	1	5	36	43
	Total	0	4	0	2	19	26	14	44	109
Observer B	3D images									
Dry bones	Phases	1	2	3	4	5	6	7	8	Total
	1	1	1							2
	2		2							2
	3			4			1			5
	4				5	1	1			7
	5					13	6			19
	6					8	17	1		26
	7				1		6	9	2	18
	8					1	1	3	25	30
	Total	1	3	4	6	23	32	13	27	109

**Table 3:** Inter-method agreement for the observers

	Cohen’s kappa						Percent agreement (%)		Wilcoxon test	
	Lowest *κ*	Highest *κ*	*p*	Mean *κ*	95% CI	*p*	0-stage tolerance	1-stage tolerance	*Z*	*p*
Observer A	0.810	0.856	< 0.001	0.836	0.780–0.892	0.423	67.9	89.9	− 1.214^a^	0.225
Observer B	0.883	0.898	< 0.001	0.892	0.851–0.934	0.730	69.7	95.4	− 1.150^b^	0.250

^a^Based on negative ranges

^b^Based on positive ranges

### Age and rib phase estimation

Observer A obtained a strong linear correlation between the estimated and Iscan phase for dry bones and 3D images (Table 4).

Observer B’s estimates showed a very strong linear correlation with the Iscan phase in dry bones. Although the *ρ* value in 3D images was lower, the correlation was considered strong (Table 4).

In both observers, the percentage of correctly classified samples was higher in dry bones than in 3D images (Table 4), following phases 8, 6, and 5 in order of frequency in both modalities.

**Table 4:** Correlation and percent agreement between observed phases and Iscan phases in dry bones and 3D images

	Rho Spearman			Percent agreement (%)	
	*ρ*	95% CI	*p*	0-stage tolerance	1-stage tolerance
Observer A					
Dry bones	0.794	0.709–0.856	< 0.001	58.7	85.3
3D images	0.691	0.574–0.780	< 0.001	54.1	82.5
Observer B					
Dry bones	0.820	0.745–0.875	< 0.001	71.6	89
3D images	0.690	0.572–0.779	< 0.001	50.5	83.5

**Fig. 2:**
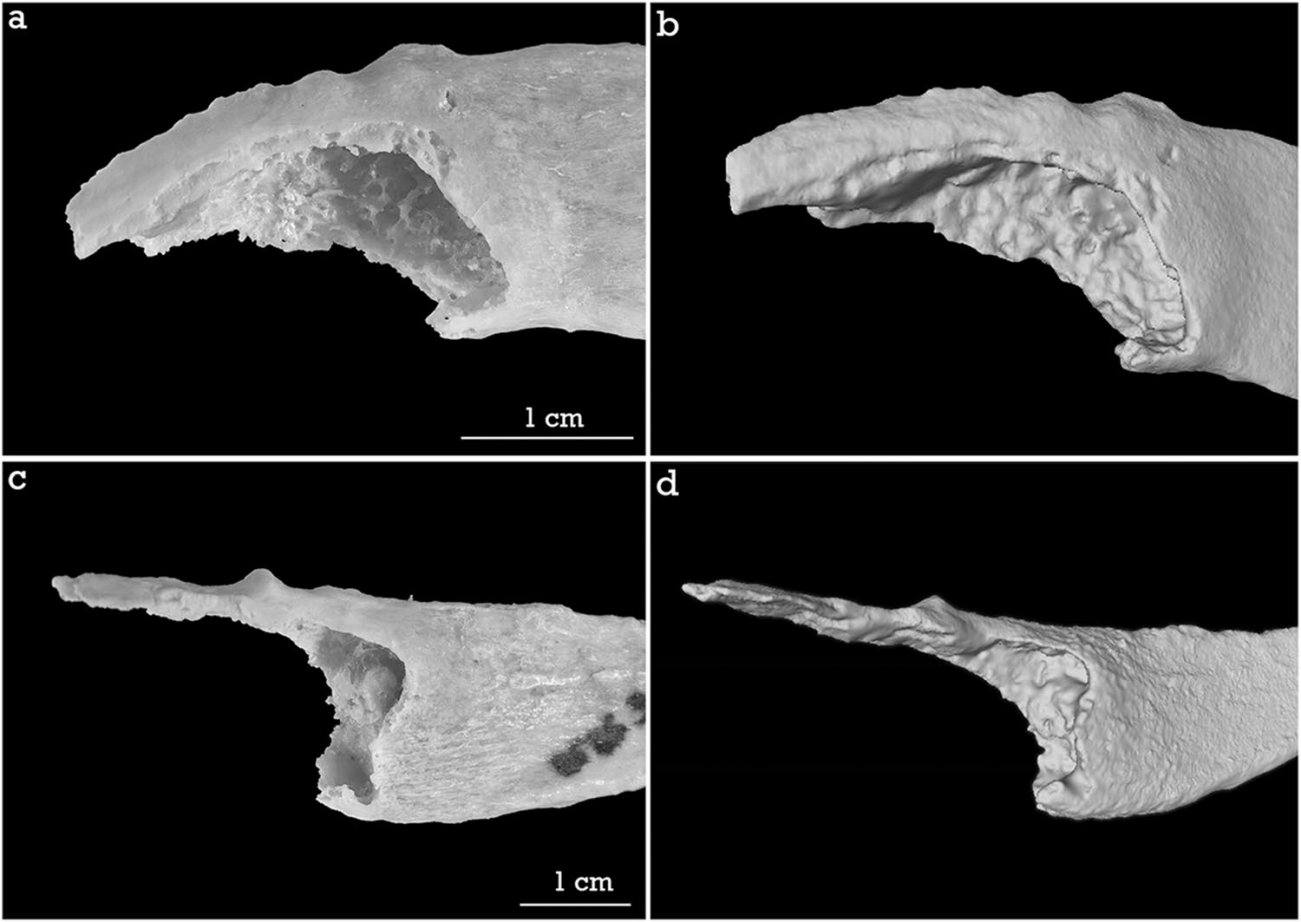
**a**–**b** Photograph and 3D image of a male aged 38 years. **c**–**d** Photograph and 3D image of a male aged 80 years (1 cm scale)

When assigning the phases, the factor of biological variability must be taken into account, since of all the characteristics valued in the sample, the quality of the bone and osteophytes is usually determinant, which can sometimes lead to error. Figure 2 a–b shows significant osteophyte development in a 38-year-old male, which is very similar to the 80-year-old shown in Fig. 2c–d. The morphological variation between the two in terms of pit depth and shape, configuration of the walls, and rim and osteophytes was not substantial, which made it difficult to estimate the younger sample. The inability to assess bone porosity and weight also constituted an important limitation in the 3D images.

**Fig. 3:**
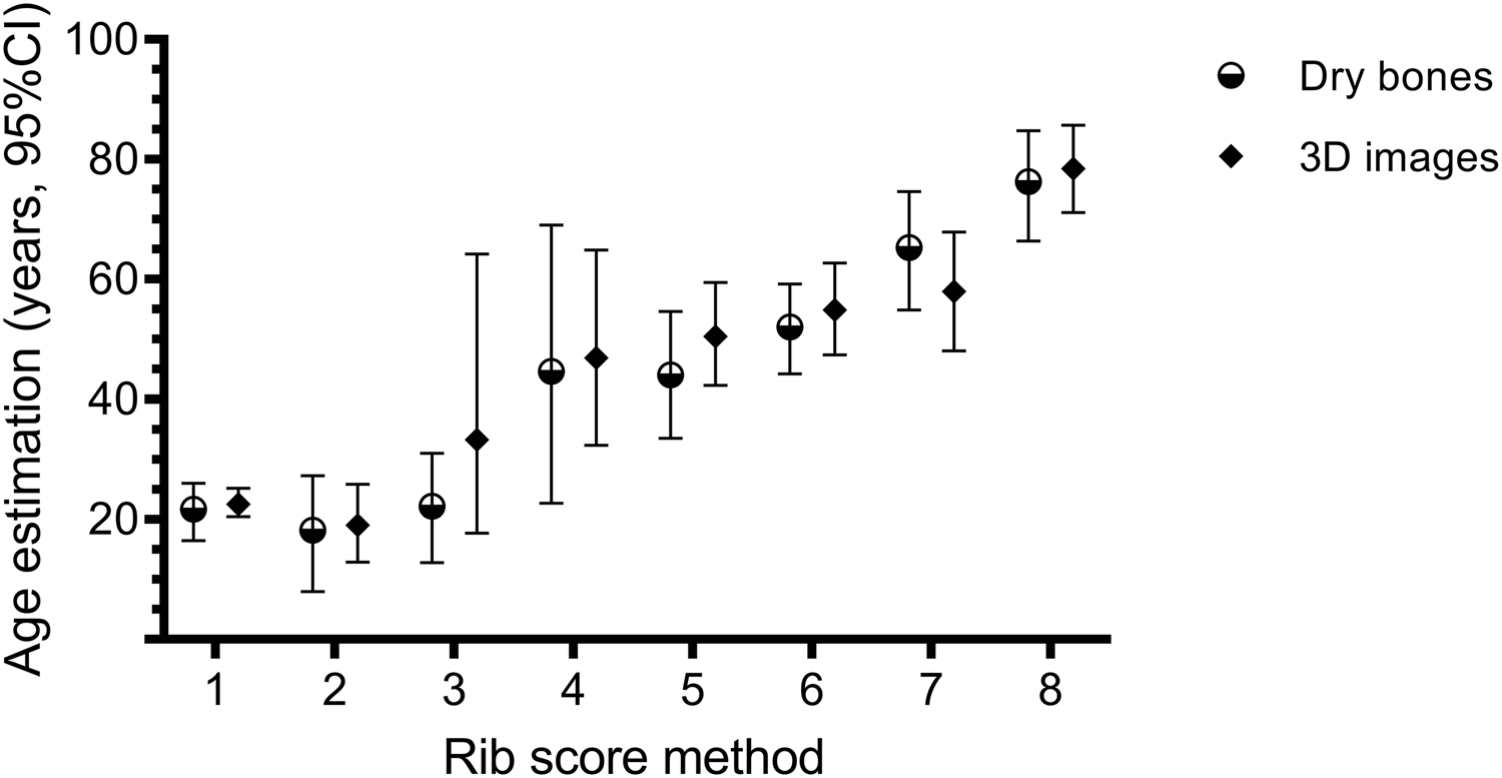
95% confidence intervals for age estimation in each phase in dry bones and 3D images

The relationship between chronological age and the estimated phase, adjusting for sex, showed statistical significance from phase 4 onwards in dry bones (Table 5) and 3D images (Table 6). The sex variable was not statistically significant in any of the modalities. The dry bone models and 3D images presented a fit of *R*^2^ = 0.65 (SEE =  ± 11.264 years) and *R*^2^ = 0.50 (SEE =  ± 13.537 years), respectively. Tables 5 and 6 and Fig. 3 show the estimated 95% CI for each model.

**Table 5:** Linear regression model of estimated age in dry bones according to phases and sex

Phase 4th rib	Mean age	Standard error	95% CI	*p*
1 (ref.)	21.68	8.049	16.472–26.015	-
2	18.18	11.264	7.949–27.281	0.757
3	22.18	9.755	12.806–31.015	0.959
4	44.07	9.205	22.739–69.057	**0.014**
5	44.62	8.427	33.540–54.666	**0.009**
6	52.04	8.222	44.249–59.215	** < 0.001**
7	65.32	8.443	54.854–74.624	** < 0.001**
8	76.30	8.244	66.376–84.831	** < 0.001**
Sex (ref. male)	+ 2.637	2.324	14.367–33.765	0.259

Bold number mean statistically significant values

**Table 6:** Linear regression model for estimated age in 3D images according to phases and sex

Phase 4th rib	Mean age	Standard error	95% CI	*p*
1 (ref.)	22.56	9.675	20.423–25.237	-
2	19.06	13.537	12.891–25.899	0.797
3	33.28	11.745	17.736–64.238	0.364
4	46.89	10.871	32.416–64.865	**0.027**
5	50.49	10.018	42.273–59.457	**0.006**
6	54.87	9.852	47.407–62.723	**0.001**
7	57.94	10.382	48.02–67.865	** < 0.001**
8	78.49	9.955	71.154–85.696	** < 0.001**
Sex (ref. male)	+ 2.881	2.818	15.34–31.872	0.755

Bold number mean statistically significant values

## Discussion

We applied the Iscan method to estimate age in a sample of 109 right ribs from a contemporary Spanish population, using samples of dry bones collected during autopsy and 3D images obtained using an EinScan Pro® structured light scanner.

Our first objective was to compare the estimates made in both types of modalities, for which we calculated the intra-observer, inter-observer, and inter-method agreements.

### Intra-observer agreement

Although certain methodological limitations existed due to high intra-observer error values [55], our agreement reached an almost perfect result both in dry bones (*κ* = 0.877–0.960) and in 3D images (*κ* = 0.954). In dry bones, the degree of agreement was very similar to that estimated in North American samples by Merritt [32] (*κ* = 0.870) and Hartnett [28], in the latter case using Spearman’s correlation coefficient (*ρ* = 0.882–1.000). It was also superior to other studies carried out in Tunisian (*κ* = 0.71–0.73) [23] and French (*κ* < 0.50) [55] populations. We also exceeded the Krippendorff alpha coefficients calculated by Dedouit et al. [42] in a French sample of dry bones (0.79) and CT scans (0.79). Other works using CT scans obtained very similar figures to those obtained by Dedouit et al. [42] and ours. Among them, two Australian studies with values of *κ* = 0.854 [43] and *κ* = 0.76 [44], and a recent study carried out in France (*κ* = 0.85) [45] are of particular importance. In our study, we used a surface scanner, so despite the results not being fully generalizable, we can confidently state that they were in line with other studies as regard the use of 3D images. Along with other previously reported data over different age estimation methods, we obtained a satisfactory level of repeatability by applying virtual anthropology techniques [48, 54, 56, 57].

### Inter-observer agreement

The highest values of agreement were found using dry bones (*κ* = 0.813), which we expected, since the method was originally developed and calibrated with physical specimens [48]. Our result is in line with similar works [28, 32–34, 42], and even higher than others [23, 55]. Fanton et al. [55] reported slight reproducibility values (*κ* < 0.20), with a limited sample size (*n* = 59), which was particularly low in the early phases.

Unlike our results, Dedouit et al. [42] showed a similar or slightly higher degree of agreement in CT reconstructions than in dry bone. Other research using CT scans with living [44, 58] and post-mortem subjects [45, 54] also shows almost perfect agreement. Although our numbers were slightly lower, they can still be considered substantial (*κ* = 0.727), and we can confirm the findings by Villa et al. [48] on pubic bone and the auricular surface regarding the superiority of direct observation of the bone (*κ* = 0.30–0.77) versus 3D visualizations. The higher resolution obtained by a surface scanner favours a more accurate reproduction of the morphological characteristics, which is why Villa et al. observed a higher inter-observer agreement (*κ* = 0.25–0.60) than with CT images (*κ* = 0.19–0.23) [48]. Other authors also state that the differences between the two modalities can be justified by the way they are operated, since surface scanner 3D models are created directly during the scanning process [59]. While there is no consensus on this point [57], comparative inferences with CT reconstructions cannot be observed in our findings.

Although the agreement values in the modalities of dry bones and 3D images were satisfactory and equivalent to other studies, the Wilcoxon test revealed significant differences in the distribution of phases for each observer. In dry bones, the medians (me) of the observers were coincident (me = 6), with any differences more evident in the 3D reconstructions (me = 6–7). However, not all the works mentioned above performed symmetry tests, so it is not possible to make comparative inferences. Fanton et al. [55] corroborate that the reproducibility of the Iscan method in dry bone is questionable (*κ* < 0.20), arguing that the descriptions of the elements to be assessed are inaccurate. Nevertheless, our agreement was almost perfect in bone and substantial in 3D images. Similarly, our inter-method agreement and the correlations of the estimated phases were almost perfect and strong, respectively. Kimmerle et al. [25] performed interobserver error analysis using various age estimation methods, including the Iscan method with dry bone. Like us, they obtained high agreement (*κ* > 0.70), especially in the most extreme phases, and usually with discrepancies of a single phase. However, they also showed highly significant differences (*p* < 0.001) among the 4 participating observers. Several factors may account for this inter-observer variation: firstly, the important morphological variety that exists in any age range; secondly, because the traits analysed at each phase undergo extremely diverse changes according to the physiological process of skeletal ageing (typically, these characteristics overlap and feature in more than one phase, since the categories are not discrete and watertight, but rather a continuum in transition [25]); and finally, because interpersonal differences in the perception of morphological traits and the interpretation of phase descriptions should be considered [20, 21]. Faced with a similar phenomenon, Mullins et al. [60] and Koterova et al. [49] stated that significant inter-observer differences do not necessarily have to affect the estimates or the applicability of the data, especially when high degrees of concordance and percentages of agreement are obtained in all the estimates. It should also be noted that this is the first study to apply the Iscan method in 3D reconstructions obtained using a structured light surface scanner in a Spanish population.

### Inter-method agreement

Virtual anthropology has become a reliable complement or alternative to the direct study of bone when estimating biological profiles using traditional anthropological methods [44, 47, 59, 61]. Our results corroborated this, as can be seen by the almost perfect agreements (*κ* = 0.836–0.892), which are higher than those obtained by Dedouit et al. [42] in CT scans (Krippendorff alpha = 0.55–0.71). In our study, we obtained slightly higher percentages of agreement with 1-stage tolerance (89.9–95.4% vs. 80.56%), although our figures for 0-stage tolerance were almost double (67.9–69.7% vs. 30.56%) [42]. The influence of observer experience on the results was not included in our study; however, in both the work of Dedouit et al. [42] and ours, the best agreements were obtained by observers with similar training (as a pathologist and forensic anthropologist) [62]. In contrast, other authors compared the two modalities for other age estimation methods and achieved fair degrees of agreement (*κ* = 0.34) [48], even with significant differences (*p* < 0.05) [57]. These authors noted that the results depend on several variables that influence the final resolution of 3D reconstructions, such as scanner resolution, contrast and brightness settings, bone volume, precision, and requirements of the estimation method for the age range used [57]. Since it is extremely difficult to control all the variables that can lead to errors when using virtual techniques [61], we thoroughly followed the recommendations for size, resolution, and post-processing [63].

The second objective of the study was to validate the Iscan method in a contemporary Spanish population using dry bones and 3D images.

### Age and rib phase estimation

The correlation with the Iscan phases was strong/very strong for dry bones (*ρ* = 0.794–0.820), in agreement with Muñoz et al. [33] (*ρ* = 0.811–0.816), Haj Salem et al. [23] (*ρ* = 0.717–0.758), and Hartnett [28] (*ρ* = 0.655–0.720), who proved the superior accuracy of the fourth rib method compared to the Suchey-Brooks pubic symphysis method. According to these figures, the results in the Spanish population were satisfactory.

In the 3D reconstructions, our results were relevant (*ρ* = 0.690–0.691), albeit slightly lower than for dry bone. In comparison, Oldrini et al. [58] (*ρ* = 0.67–0.71) and Richard et al. [45] (*ρ* = 0.72) obtained equivalent results in CT scans, while Dedouit et al. [42] showed no significant differences in the percentages according to 0-stage tolerance between the two modalities (58.3% in bone; 63.9% in CT scans), which were similar to ours (58.7–71.6% in dry bones; 50.5–54.1% in 3D images). The figures were also very similar in the studies conducted by Merritt in 2014 [32] and 2018 [43] (57.5% in bone; 57.9% in CT scans, respectively). We must also consider that the use of 3D reconstructions limits the analysis of certain bone details typically found in tactile examination, such as weight, bone quality [17, 18, 28], or porosity. In addition, the assessment may be subject to lower precision due to the method used in 3D images, the observer’s experience with virtual anthropology, and the configuration of the scanner itself [64]. Villa et al. [48] recommend the use of quantitative analysis to reduce observer subjectivity, a technique used by Nikita in 2013 [65] on an archaeological sample in the UK, without obtaining satisfactory results in the estimation of age. On the other hand, although our correlations were better in dry bones, the percentages of agreement with 1-stage tolerance in both modalities were over 80%. These findings were consistent with the high values of inter-method agreement, so the method could alternatively be applied to 3D images in the Spanish population.

After the age of 50, maturational changes in the rib are less evident, which therefore minimizes the differences between age groups [23] and reduces the percentages of correct classification [32]. Haj Salem et al. [23] presented better agreement percentages in the first four phases in a Tunisian male sample. Our results were better in phases 8 and 6, in line with Oldrini et al. [58] from the age of 60 onwards. These findings show that the composition of the sample is a determining factor, since a significant proportion of the subjects used when Iscan et al. developed this method [17, 18] were over 50 years old (30% males, 40% females). In our sample, this figure was even higher, with 65.1% over the age of 50. However, we must also consider that the characteristics described for each phase are not mutually exclusive, which makes it easier to estimate the extreme phases than the intermediate ones [25, 33].

Despite achieving adequate correlation and classification values, the Iscan method tended to overestimate age in the early phases (1–3) of our sample and underestimate it in the latter (4-8) in dry bones. A similar phenomenon is described in the literature [32, 42, 44, 66], with underestimation of age being more common in the more advanced phases [23, 31, 33, 34, 67]. In our case, this could have been caused by several factors, including the low number of subjects in the early phases, the mimicry of Iscan et al. age groups with our sample, or that the age limit in their work (85 and 90 years in males and females, respectively) was below our maximum age (7.3% of subjects above) [17, 18]. Genetic and environmental differences between populations are also an added factor [31, 33], with lower precision reported in samples of African American [24], South African [68], or Southeast Asian origin (*ρ* = 0.268–0.565) [67]. In addition, Merritt [69] demonstrated the influence of body mass index (BMI), noting a systematic underestimation of age in low BMI subjects.

A similar trend was evident in the 3D images, except for a slight variation in phase 3 (where age was underestimated) and in phase 7 (where age was overestimated). It is important to note that the use of the scanner adds a series of limitations inherent to the technique. For instance, surface scans have a high resolution, but they tend to reduce bone surface irregularities at the edges, so in more advanced phases this may lead to the mistaken choice of a younger phase. In addition, it is unable to assess wall thickness well and does not permit bone porosity and quality to be measured [42, 64]. Russell et al. [70] also pointed out that the descriptions of the Iscan phases are not accurate in terms of bone weight and quality. In fact, many of the characteristics associated with elderly individuals can only be appreciated by the tactile perception of bone to assess its porosity, brittleness, and relative weight, and great importance has been attached to these characteristics, as they are a decisive factor in the choice of phases, especially among the most advanced ones [28, 71]. This was one of the most important limitations in our experience with 3D imaging, and a clear example of this is shown in Fig. 2. Given the impossibility of evaluating porosity and fragility, Merritt [43] proposed the study of trabecular and cortical bone quality in CT scans, obtaining good results. However, this option is not possible for the surface scanner as it can only capture external surfaces that are within its field of vision [47].

All these biological and technical factors resulted in the regression models calculated in bone (*R*^2^ = 0.65) and 3D image (*R*^2^ = 0.50) being moderate. Richard et al. [45] (*R*^2^ = 0.58) reported similar values and Blaszkowska et al. [44] (*R*^2^ = 0.65) slightly higher with CT scans, which shows the limitations of these models in predicting age. Our SEE value in bone was higher than those reported by Macaluso and Lucena [72] in a Spanish population (± 11.264 vs. ± 8.27–8.32 years), although they applied Verzeletti et al. fourth rib component method [27]. Our SEE value in 3D images was also superior to that of Blaszkowska et al. [44] by 2.3 years (± 13.5 vs. ± 11.2 years). Unlike Richard et al. [45], our estimates were more accurate in phases 6 and 8 (SE bones: ± 8.222–8.244; 3D images: ± 9.852–9.955 years). Furthermore, the small number of subjects contained in the first four phases (*n* = 13) was a determining limiting factor, despite being contradictory data because, in younger subjects, more important morphological changes occur and at a greater speed. SE values were lower in dry bones than in 3D reconstructions, although the maximum variations between the two models were 2.3 years (phase 2). We obtained SEE and SE high, but in no case did they reach ± 15 years [73]. In line with other works [28, 44, 45], the 95% CIs were much broader than those of Iscan et al. [17, 18], reaching maximum figures of up to 46.5 years (in phases 4 and 3 of dry bones and 3D images) and a significant overlap between the phases.

In view of the results of this study, to achieve a more precise estimation of age in a Spanish population, it would be beneficial to carry out a multicentre study, which would allow the number of subjects in each age group and sex to be increased within a reasonable time period. Having a good balance between groups would enable us to carry out a transition analysis and obtain reference standards of the age at which the change from one phase to another occurs in a Spanish population, in order to compare it with other studies. The rise of virtual anthropology techniques has paved the way for the comparison with post-mortem CT scans, which, although more detailed post-processing is needed, do not require skeletonization of the sample. The costs are higher; however, it is a non-invasive technique that can be performed simultaneously with a medical-legal autopsy [74]. On the other hand, quantitative analyses based on morphometric geometric [75] and multiple mathematical methods [76] also play an important role in forensic anthropology, as well as machine learning approaches [77, 78], which have not been used to date with the fourth rib method. Finally, our results point to the traditional international recommendation to evaluate the Iscan method together with other age estimation indicators in adults [8, 62].

### Limitations of the study

Our sample size was limited, due to the difficulty in obtaining forensic samples. The disproportion between males and females reflects the usual casuistry in medical-legal autopsies (~ 78.6/21.4%) [79]. Even so, the observers arrived at their estimations using the reference casts, according to the sex of the subject. In addition, although the linear regression models were adjusted to the sex variable, they did not produce a significant result. A similar problem was found in the variations of the different age groups of the sample, since there is a clear predominance of subjects of 40–50 years of age (> 60%) in legal autopsies. Due to this limitation, it was not possible to perform a transition analysis between the phases.

The difficulty in evaluating the porosity and quality of the bone in the 3D images of the surface scanner was also a limitation for achieving a more precise estimate, especially in the more advanced phases (7–8).

## Conclusions

The last few years have seen an increase in the use of virtual anthropology techniques to estimate the parameters of a subject’s biological profile in forensic identification. Of these, age appears to be one of the most difficult parameters to assess, due to its wide variability. This study is the first work to compare the application of the Iscan method in dry bones and 3D reconstructions obtained using a surface scanner in a contemporary Spanish population. The intra-observer, inter-observer, and inter-method agreements confirmed the use of 3D imaging as a valid and reliable alternative. However, it must be considered that the original method was developed using real bones and that the estimation with 3D reconstructions varies depending on the operator and the limitations of the technique, which most affects characteristics such as weight and bone quality. The present study showed good correlations when estimating the Iscan phases in a Spanish population, but the age of the subjects was overestimated in the earlier phases and underestimated in the later ones. The possible population differences produced sizeable error values and confidence intervals, so we would therefore recommend the joint use of different methods and analysis of multiple anatomical sites.
